# Supplementary material for: Functional Characterization of a Familial ALS-Associated Missense TBK1 (p-Arg573Gly) Mutation in Patient-Derived Lymphoblasts
Source: Int J Mol Sci. 2023 Feb 2;24(3):2847. doi: 10.3390/ijms24032847 (PMC9917786; doi:10.3390/ijms24032847)
Supplement: Supplementary file 1 [file ijms-24-02847-s001.zip › ijms-2136075-supplementary.pdf]

A

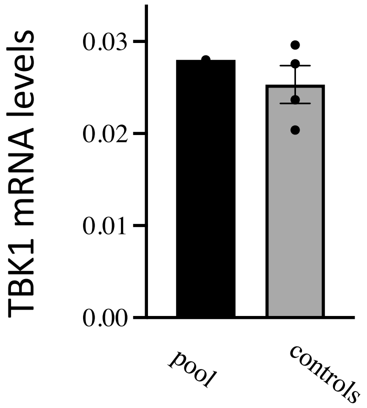

B

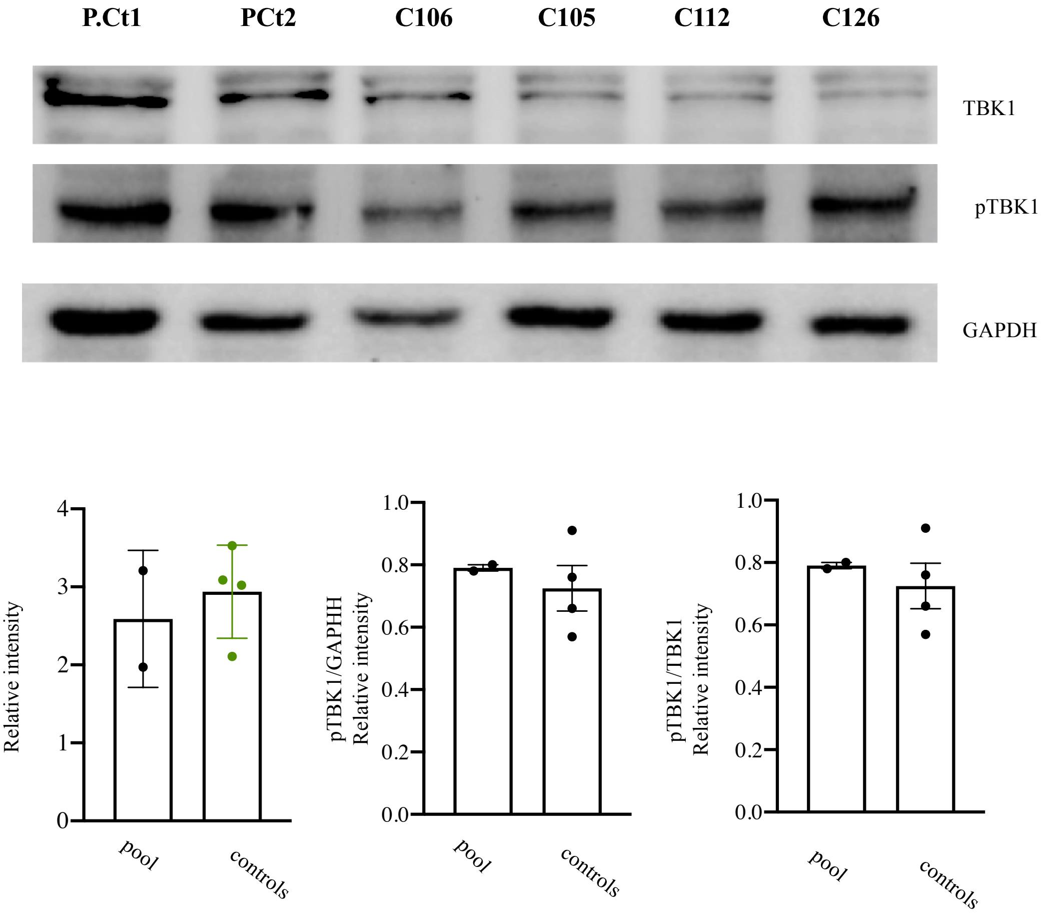

Supplementary Figure S1. TBK1 expression levels in human lymphoblasts.
Lymphoblasts from four control subjects were incubated individually or pooled at the initial density of $1x10^6$ cells x ml^{-1} in RPMI medium containing 10%FBs for 24 h. Then cells were harvested to isolate RNA and to prepare extracts for WB. A) TBK1mRNA levels were analyzed by quantitative RT-PCR. Data represent the mean ± SEM. B) TBK1 protein content. Densitometric analysis are the mean ± SEM. PCt1 and Pct2 are two doferent preparations of pooled control cell lines
